# Risk stratification and prediction of severity of COVID-19 infection in patients with preexisting cardiovascular disease

**DOI:** 10.3389/fmicb.2024.1422393

**Published:** 2024-07-25

**Authors:** Stanislava Matejin, Igor D. Gregoric, Rajko Radovancevic, Slobodan Paessler, Vladimir Perovic

**Affiliations:** ^1^Department of Advanced Cardiopulmonary Therapies and Transplantation, University of Texas Health Science Center at Houston, Houston, TX, United States; ^2^Institute for Human Infections and Immunity, University of Texas Medical Branch, Galveston, TX, United States; ^3^Laboratory of Bioinformatics and Computational Chemistry, Institute of Nuclear Sciences Vinca, National Institute of the Republic of Serbia, University of Belgrade, Belgrade, Serbia

**Keywords:** COVID-19, SARS-CoV-2, cardiovascular diseases, machine learning, prediction of survival

## Abstract

**Introduction:**

Coronavirus disease 2019 (COVID-19) caused by SARS-CoV-2 is a highly contagious viral disease. Cardiovascular diseases and heart failure elevate the risk of mechanical ventilation and fatal outcomes among COVID-19 patients, while COVID-19 itself increases the likelihood of adverse cardiovascular outcomes.

**Methods:**

We collected blood samples and clinical data from hospitalized cardiovascular patients with and without proven COVID-19 infection in the time period before the vaccine became available. Statistical correlation analysis and machine learning were used to evaluate and identify individual parameters that could predict the risk of needing mechanical ventilation and patient survival.

**Results:**

Our results confirmed that COVID-19 is associated with a severe outcome and identified increased levels of ferritin, fibrinogen, and platelets, as well as decreased levels of albumin, as having a negative impact on patient survival. Additionally, patients on ACE/ARB had a lower chance of dying or needing mechanical ventilation. The machine learning models revealed that ferritin, PCO2, and CRP were the most efficient combination of parameters for predicting survival, while the combination of albumin, fibrinogen, platelets, ALP, AB titer, and D-dimer was the most efficient for predicting the likelihood of requiring mechanical ventilation.

**Conclusion:**

We believe that creating an AI-based model that uses these patient parameters to predict the cardiovascular patient’s risk of mortality, severe complications, and the need for mechanical ventilation would help healthcare providers with rapid triage and redistribution of medical services, with the goal of improving overall survival. The use of the most effective combination of parameters in our models could advance risk assessment and treatment planning among the general population of cardiovascular patients.

## 1 Introduction

Coronavirus disease 2019 (COVID-19), caused by severe acute respiratory syndrome coronavirus 2 (SARS-CoV-2), is a highly contagious viral disease (Cascella et al., 2024). The initial documented cases were reported in Wuhan, Hubei Province, China, in late December 2019. SARS-CoV-2 quickly spread worldwide, prompting the World Health Organization (WHO) to declare it a global pandemic on March 11, 2020 (Cascella et al., 2024). Since then, COVID-19 has remained a significant cause of morbidity and mortality on a global scale (Raisi-Estabragh et al., 2023).

SARS-CoV-2 infection results from the attachment of the viral surface spike protein to the human angiotensin-converting enzyme 2 (ACE2) receptor after activation of the spike protein by transmembrane protease serine 2 (Clerkin et al., 2020; Vosko et al., 2023). ACE2 is prominently expressed in the heart and plays a crucial role in counterbalancing the effects of angiotensin II, especially in conditions marked by excessive activation of the renin-angiotensin system, such as hypertension (HTN), congestive heart failure (CHF), and atherosclerosis (Clerkin et al., 2020). The interaction between the viral spike protein and ACE2, which initiates the virus’s entry into host cells, may contribute to the cardiovascular manifestations of COVID-19, in addition to the impacts of respiratory infection and inflammation (Nishiga et al., 2020). Meanwhile, it is well established that preexisting cardiovascular diseases (CVDs) significantly elevate the risk of severe and potentially fatal outcomes in COVID-19 (Clerkin et al., 2020; Vosko et al., 2023).

The clinical manifestation and progression of COVID-19 vary significantly, encompassing asymptomatic or mild symptoms (such as fever, dry cough, and fatigue) to severe conditions like severe pneumonia and acute respiratory distress syndrome (ARDS), which may lead to a potentially fatal outcome (Italia et al., 2021). SARS-CoV-2 can also induce acute myocardial injury along with long-term damage to the cardiovascular system (Wang et al., 2020). Pre-existing CVD appears to be associated with more adverse outcomes and an elevated risk of death among COVID-19 patients (Nishiga et al., 2020). Additionally, COVID-19 itself can lead to myocardial injury, arrhythmia, acute coronary syndrome, and venous thromboembolism (Nishiga et al., 2020). Numerous studies have established a connection between exposure to COVID-19 and an increased likelihood of experiencing adverse cardiovascular outcomes, persisting even after recovery from the acute illness (Raisi-Estabragh et al., 2023).

Heart failure (HF) in the context of COVID-19 introduces a distinct set of challenges that can complicate the way it is presented and managed, as well as its overall prognosis (Bader et al., 2021). The management of HF has been adversely affected by the COVID-19 pandemic, resulting in decreased hospitalizations due to the closure of medical facilities or restricted access to healthcare services. The measures implemented during the pandemic have led to a decrease in the overall number of hospitalizations, subsequently contributing to an elevated mortality rate in HF, likely exacerbated by the lack of available care (Italia et al., 2021). It is established that individuals admitted to the hospital for COVID-19 may experience both an acute worsening of pre-existing HF and the development of new-onset HF, attributable to myocardial injury and complications affecting the cardiovascular system (Italia et al., 2021). Having pre-existing HF is identified as a risk factor for a more severe clinical course of COVID-19 and serves as an independent predictor of in-hospital mortality (Italia et al., 2021). Certain studies have indicated that among the population of COVID-19 patients who were hospitalized, the prevalence of HF ranged from 4 to 21% (Italia et al., 2021). Additionally, the hospitalization of COVID-19 patients with pre-existing HF in the year 2020 was independently linked to an elevated risk of mortality (Italia et al., 2021).

A retrospective analysis revealed that HF was connected to an increased risk of both mechanical ventilation and mortality among patients hospitalized for COVID-19, irrespective of left ventricular ejection fraction (LVEF) (Alvarez-Garcia et al., 2020). Consistent findings were observed in an Italian multicenter study, where HF emerged as an independent predictor of mortality and a risk factor for various in-hospital complications, including acute HF, acute renal failure, and multiorgan failure (Tomasoni et al., 2020). Hence, a thorough comprehension of the hemodynamic and diagnostic implications is crucial for the proper triage and management of these patients (Bader et al., 2021).

To effectively triage and manage COVID-19 patients with preexisting CVD, thus reducing the risk of requiring mechanical ventilation and enhancing survival, we believe it is essential to evaluate potential markers indicating the severity of the COVID-19 infection. Irregular cardiac biomarkers are frequently observed in COVID-19 and can arise from various mechanisms, including viral entry through ACE2 receptors, direct cardiac injury, increased thrombotic activity, stress cardiomyopathy, etc. (Bader et al., 2021). As an illustration, myocardial injury may occur due to the associated cytokine storm, evident through heightened levels of interleukin-6 (IL-6), ferritin, lactate dehydrogenase (LDH), and D-dimer, or from the direct impact of SARS-CoV-2 on the heart (Clerkin et al., 2020; Vosko et al., 2023).

Advanced age is a significant predictor of mortality in patients with COVID-19 (Gallo Marin et al., 2021). Additionally, data indicate that male sex is a factor independently associated with the severity of COVID-19 (Gallo Marin et al., 2021). Pre-existing conditions, including CVD, chronic kidney disease, chronic lung diseases, diabetes mellitus, HTN, immunosuppression, obesity, and sickle cell disease, predispose patients to an unfavorable clinical course and an increased risk of intubation and death in the context of COVID-19 (Gallo Marin et al., 2021). A body mass index (BMI) exceeding 30 is deemed a robust predictor of an adverse outcome in the context of COVID-19 (Gallo Marin et al., 2021).

Increased levels of glycosylated hemoglobin (HbA1c) have been correlated with inflammation, hypercoagulation, and elevated mortality. Findings consistently associated with poorer outcomes include heightened levels of D-dimer, C-reactive protein (CRP), and high-sensitivity cardiac troponin I (Gallo Marin et al., 2021). Increases in aspartate aminotransferase (AST) and alanine aminotransferase (ALT) are more likely to occur in patients with severe or critical cases of COVID-19 and are indicative of end-organ damage (Gallo Marin et al., 2021). Fibrinogen levels have been shown to be elevated in patients with severe COVID-19 disease (Guevara-Noriega et al., 2020). Furthermore, observing elevated levels of ferritin is a significant finding in COVID-19 and is associated with an increased risk of mortality (McMillan et al., 2021). Abnormalities in markers of cellular injury, notably elevated LDH, have been correlated with increased disease severity and serve as important predictors of respiratory failure in patients with COVID-19 (Gallo Marin et al., 2021). Furthermore, a recent study indicates that COVID-19 may be linked to both systolic and diastolic left ventricular (LV) dysfunction, along with the most common echocardiographic findings such as LV diastolic impairment, pulmonary hypertension, and right ventricular dysfunction (Italia et al., 2021).

In our paper, we proposed to evaluate individual parameters that could potentially predict outcomes such as death and increased risk of complications with a higher risk of needing mechanical ventilation in our cardiovascular patient population. Since our study was designed and conducted during the early COVID-19 pandemic, we included proportionally the same number of COVID-19 positive and COVID-19 negative patients, both without prior history of COVID-19 infection or vaccination against COVID-19. Furthermore, we seek to describe how the combination of multiple parameters, including COVID-19 infection, could influence the outcomes.

Our objective is to describe potential markers that could correlate with higher survival chances in our cardiovascular patients with HF. Therefore, we tested the association between these markers and the severity of COVID-19 infection, including survival and the likelihood of need for mechanical ventilation. We believe our work will expand our understanding of the biological processes of COVID-19 infection in cardiovascular patients and allow easier and faster identification of patients with a higher risk of needing mechanical ventilation. Furthermore, we seek to expand our research and focus on cardiovascular patients in general and learn how to effectively and quickly predict the likelihood of the outcomes, identify associated risk factors, and help healthcare providers generate the most effective and time-efficient management and treatment strategy for their patients.

## 2 Materials and methods

### 2.1 Patients’ data

This is a prospective, observational, single-center study designed to collect baseline de-identified blood samples and clinical data on hospitalized cardiovascular patients to better evaluate the correlation between these parameters and patients’ outcomes. Hospitalized patients from January 2021 to May 2021 were included in the study (*n* = 60). They were classified into two categories based on their COVID-19 status: 30 with proven COVID-19 infection during the acute phase of the disease and 30 patients with no history of COVID-19 infection as a comparable group. None of the patients reported a previous COVID-19 infection and have not received the vaccine yet. COVID-19 infections were confirmed using a polymerase chain reaction (PCR) COVID-19 test.

After obtaining IRB approval (IRB number HSC-MS-20-1209) to conduct the study, we collected baseline data to serologically screen patients to detect antibodies against SARS-CoV-2 antigen to help us better characterize the serological status of patients and potential previous exposures. We used virological and serological assays to further test the deidentified blood samples for the presence of SARS-CoV-2. A neutralization assay and a commercially available ELISA were used to identify the presence of the anti-SARS-CoV-2 antibody. In addition to serologic analyses, we collected baseline clinical data (such as demographic characteristics, medical history, laboratory data, echocardiographic findings, etc.) from patients’ electronic medical records to compare the serologic findings with their clinical presentation and to better asses their response based on the COVID-19 status and as well as the outcome. We identified patients by their MRNs (medical record numbers) and kept a separate password-protected document with their personal information.

### 2.2 Statistical analyses

For statistical analyses, we used the Fit an Analysis of Variance Model and Student’s two-sample t-test (Kim, 2015; Mishra et al., 2019) within the “Stats” package from the R Statistical Software v4.3.0 (R Core Team, 2023; RStudio Team, 2023). We performed ANOVA and t-tests to assess the statistical significance of an association between patients’ parameters and patients’ survival as well as the likelihood of the need for mechanical ventilation. We conducted three separate analyses: (1) excluding pairs with missing data, (2) replacing missing data with average values, and (3) replacing missing data with reference values.

### 2.3 Machine learning

Furthermore, to establish the correlation between the combination of individual parameters and predictive outcomes, we used machine learning techniques. The problem of predicting patients’ survival or the need for mechanical ventilation is a binary classification problem. To generate machine learning (ML) predictors, we used an Ensemble model composed of several base classifiers using the following ML algorithms: Random forest (RF) (Breiman, 2001; Geurts et al., 2006; Parmar et al., 2019), the generalized linear model (GLM) (Dobson and Barnett, 2018; Nykodym et al., 2024), Gradient Boosting Machine (GBM) (Friedman, 2001; Natekin and Knoll, 2013; Malohlava and Candel, 2024), and Deep learning (DL) (LeCun et al., 2015; Goodfellow et al., 2016; Candel and LeDell, 2024). The ML ensemble models were generated using the H2O.ai platform v3.42.0.1 (LeDell et al., 2023; H2O.ai, 2024).

**Figure 1:**
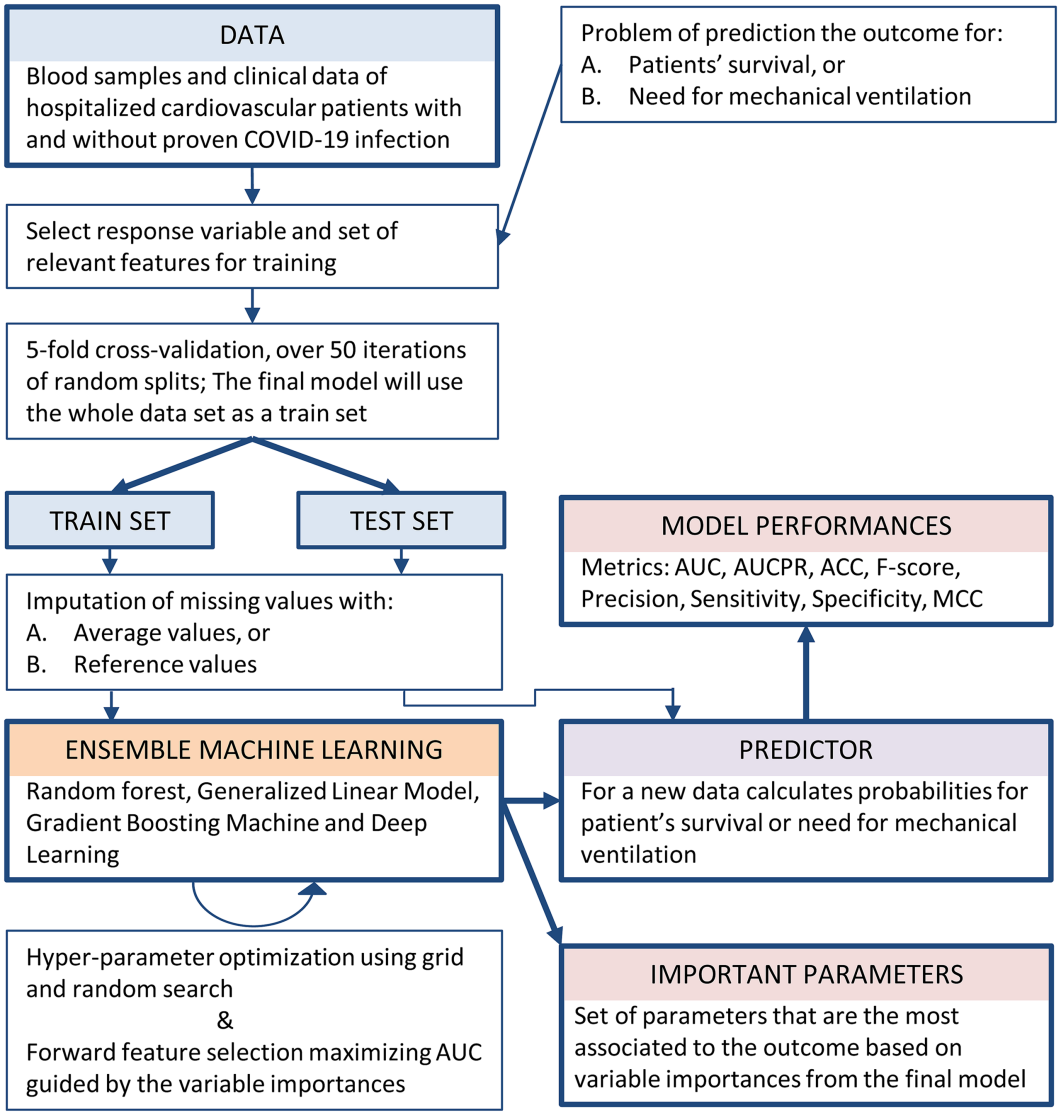
Workflow of the machine learning process for the creation and evaluation of AI-based models for predicting both patients’ survival and the need for mechanical ventilation.

The entire machine learning process for creating and evaluating AI-based models for predicting patients’ survival and the need for mechanical ventilation is presented as a workflow scheme in Figure 1.

As performance metrics, we used the area under the receiver operating characteristic curve (AUC), the area under the precision-recall plots (AUCPR), accuracy (ACC), precision, sensitivity (or recall), specificity, F score, and the Matthews correlation coefficient (MCC). The equations for calculating the performance metrics are provided in the Supplementary Document.

The evaluation of ML predictors was carried out on 5-fold cross-validation, with 50 iterations of random splits, where performance metrics were calculated as the average over all iterations. For the calculation of the metrics, thresholds were selected based on the maximization of the F-score on predicted probabilities for each train set, i.e., each split in the cross-validation procedure. To correctly evaluate the ensemble model, the same splits were used for all submodels in the ensemble. The splitting procedure utilized stratified folding based on the response variable (LeDell et al., 2023).

The feature selection procedure was performed using forward feature selection, maximizing AUC, guided by the variable importance scores. In other words, features with higher importance were selected first. The variable importance of each feature for the ensemble was calculated as the average of the variable importances from the included ML submodels, depending on the machine learning algorithm: the relative influence of each variable for tree-based algorithms, coefficient magnitudes for GLM, or the weights connecting the input features to the first two hidden layers for DL, as implemented in the H2O.ai platform (LeDell et al., 2023).

To identify the important parameters, i.e., the set of parameters that are most correlated to the outcome, we used the calculation of “variable importances” from the final Ensemble model. This allows us to measure their synergistic effect on the outcome. Through feature selection and variable importance techniques, the final Ensemble model comprises attributes that work together in the best way to predict the outcome, i.e., have the most influence on the outcome. Our goal was to identify important parameters that, in combination with COVID-19 infection, are most correlated to the outcomes. For machine learning, we used the imputation of missing values with (1) average values and (2) reference values.

## 3 Results

Our data analysis included 60 patients with an average age of 56.87 ± 14.78. Thirty-six were males and 24 were females. Thirty patients were diagnosed with proven COVID-19 infection, and 30 tested negative for COVID-19 infection. The patient data collected for analysis is provided in the Supplementary Data File.

### 3.1 ANOVA and *t*-test analyses

To perform the analysis, we compared the patient’s parameters with two outcomes: death and severe complications requiring mechanical ventilation. Both ANOVA and two-tailed t-test showed similar results (Tables 1, 2). By excluding pairs with missing data or by replacing missing data with average values, we obtained similar results (Tables 1, 2 and Supplementary Figures S1, S2).

**Table 1:** The ANOVA and *t*-test analyze the relationship between each parameter and patient survival.

**Parameter**	**ANOVA *F*-value**	**ANOVA *p*-value**	**t-test t-stat**	**t-test p-value two-tail**
Mechanical ventilation *	54.0000	7.61E-10	6.8963	1.65E-07
Albumin *	23.6422	9.23E-06	−4.9494	2.06E-05
AB titter binary *	22.7886	1.27E-05	4.7165	4.71E-05
COVID-19 infection *	20.3000	3.26E-05	5.2425	3.75E-06
ECMO *	15.7354	2.03E-04	3.3398	2.82E-03
Ferritin *	16.2624	2.28E-04	3.2811	4.30E-03
ACE/ARB *	9.6667	2.91E-03	−4.7726	2.32E-05
AB titter *	9.4617	3.20E-03	2.2626	3.54E-02
Platelets *	8.2195	5.80E-03	−2.9858	5.07E-03
Sex *	6.2462	0.0153	2.7906	7.87E-03
Fibrinogen *	5.5965	0.0225	2.1887	0.0376
O2Sat	2.9949	0.0916	−1.6215	0.1184
LVEDD	2.1057	0.1526	−1.4622	0.1527
ALP	1.9291	0.1703	1.3501	0.1869
AST	1.5338	0.2205	0.8718	0.3945
D-dimer	1.4237	0.2389	1.1869	0.2432
ALT	1.3991	0.2417	0.8461	0.4082
PCO2	1.1837	0.2845	1.0896	0.2838
HTN	0.9553	0.3324	−0.9016	0.3752
CKD	0.9355	0.3375	0.9083	0.3714
BMI	0.7281	0.3970	−0.9554	0.3448
FIO2	0.7206	0.4034	0.8523	0.4018
BNP	0.5183	0.4757	−1.1485	0.2582
LVEF	0.4990	0.4829	0.7197	0.4766
DM	0.3954	0.5320	0.6154	0.5428
LDH	0.3829	0.5402	0.5777	0.5696
CRP	0.2664	0.6100	0.5184	0.6085
Troponin I	0.0875	0.7689	−0.3412	0.7347
Age	0.0642	0.8008	0.2429	0.8097
HbA1c	0.0361	0.8505	−0.2162	0.8305
PEEP	0.0146	0.9050	−0.1274	0.9006
RV dysfunction	0.0078	0.9299	0.0879	0.9305
COPD	0.0074	0.9315	−0.0868	0.9314
PO2	0.0016	0.9687	0.0396	0.9686

Pairs with missing data are excluded from the analysis. The table is sorted by ANOVA *p-*value in ascending order. Statistically significant parameters with both *p* values less than 0.05 are marked with an asterisk.

**Table 2:** The ANOVA and *t*-test analyses between each parameter and the likelihood of patients needing mechanical ventilation.

**Parameter**	**ANOVA *F*-value**	**ANOVA *P*-value**	***t*-test *t*-stat**	***t*-test *p*-value two-tail**
ECMO *	116.0000	1.85E-15	−7.5498	3.92E-07
Survival outcome *	54.0000	7.61E-10	6.2545	1.27E-06
Albumin *	43.5922	1.36E-08	6.7933	3.18E-08
AB titter binary *	33.6583	2.89E-07	−5.6764	1.88E-06
COVID-19 infection *	27.2941	2.48E-06	−5.9639	2.04E-07
Fibrinogen *	23.2229	1.75E-05	−4.4523	1.26E-04
AB titter *	20.9045	2.58E-05	−3.3908	2.82E-03
ALP *	16.8629	1.30E-04	−2.9316	8.52E-03
Ferritin *	17.0152	1.71E-04	−3.6380	1.68E-03
D-dimer *	12.3554	1.00E-03	−3.0826	5.32E-03
ACE/ARB *	11.6000	1.20E-03	4.8374	2.09E-05
Platelets *	10.0169	2.49E-03	3.3166	1.84E-03
CRP *	10.6683	2.96E-03	−3.4539	1.99E-03
PCO2 *	8.5223	6.28E-03	−2.9319	6.30E-03
Age *	5.3394	0.0244	2.3140	0.0261
FIO2 *	5.3661	0.0284	−2.3529	0.0278
LVEDD *	4.8536	0.0320	2.3058	0.0258
LVEF *	4.7322	0.0339	−2.3383	0.0236
ALT	4.5870	0.0364	−1.5514	0.1366
HTN	4.3866	0.0406	1.8509	0.0747
AST	3.8541	0.0544	−1.3987	0.1777
O2Sat	3.8029	0.0586	1.9501	0.0598
LDH	3.5744	0.0672	−1.8906	0.0745
PEEP	2.0316	0.1688	−1.9780	0.0878
DM	1.7193	0.1950	−1.2769	0.2099
CKD	1.5890	0.2125	1.3639	0.1791
COPD	1.2782	0.2629	1.3166	0.1934
Sex	1.2340	0.2712	−1.1372	0.2621
HbA1c	0.8912	0.3522	−0.8710	0.3943
BNP	0.6072	0.4404	1.2414	0.2222
RV dysfunction	0.4140	0.5226	−0.6414	0.5250
PO2	0.1838	0.6710	0.4225	0.6758
BMI	0.1112	0.7400	−0.3587	0.7214
Troponin I	0.1073	0.7449	0.3653	0.7169

Pairs with missing data are excluded from the analysis. The table is sorted by ANOVA *P-*value in ascending order. Statistically significant parameters with both *p-*values less than 0.05 are marked with an asterisk.

#### 3.1.1 ANOVA and *t*-test analyses between each parameter and the patient’s survival

Our data showed that a positive antibody titer against the SARS-CoV-2 antigen, as well as current COVID-19 infection, correlates with an increased risk of mortality among our study sample. Males were more likely to die than females. As expected, those who were on extracorporeal membrane oxygenation (ECMO) or mechanical ventilation had an increased risk of mortality. Among the laboratory parameters, our study data showed a negative correlation between levels of fibrinogen and ferritin and survival. Patients with a lower platelet count and albumin levels were more likely to die. Those who were on ACE/ARB medication had higher survival rates. Compared to other similar studies, our data did not show a correlation between patients’ age, echocardiographic characteristics, or preexisting comorbidities and increased risk of mortality. By replacing missing data with average values, the results were very similar. Additionally, D-dimer, C-reactive protein (CRP), and partial pressure of carbon dioxide (PCO2) have been shown to positively correlate with patients’ survival.

#### 3.1.2 ANOVA and *t*-test analyses between each parameter and the patient’s likelihood of needing mechanical ventilation

By excluding comparing pairs with missing data or by replacing missing data with average values, a positive correlation was found between the presence of antibodies against SARS-CoV-2 antigen and current COVID-19 infection with the need for mechanical ventilation due to severe complications. Increased age and use of ACE/ARBs were shown as protective factors for mechanical ventilation. Similarly, decreased platelet count and albumin levels were found to correlate with an increased risk for the need for mechanical ventilation when estimating the risk of survival. Furthermore, increased levels of D-dimer, Fibrinogen, CRP, Ferritin, ALP, ALT, and PCO2 were associated with an increased risk for the need for mechanical ventilation. Echocardiographic criteria such as Left ventricular ejection fraction (LVEF) and Left ventricular end-diastolic volume (LVEDD) were also found to be directly correlated with the outcome. Therefore, careful laboratory and echocardiographic evaluation could guide proper management and treatment of cardiovascular patients and help predict the risk for patients. Additionally, a history of HTN was identified as a good predictor of severe complications in cardiovascular patients needing mechanical ventilation. The results remained the same when replacing variables with average values, with the addition of LDH as a predictor of the outcome.

### 3.2 Machine learning in the identification of important parameters

The full list of 35 features used for generating machine learning models for predicting a patient’s survival and likelihood of ending up on mechanical ventilation, along with the average values and reference values for each feature used for imputation of missing data, is provided in Table 3. For some parameters, reference values are not provided in the table, as they had no missing values in the data.

**Table 3:** List of patients’ parameters with average and reference values.

**Patient’s parameter**	**Description**	**Average**	**Reference-male**	**Reference-female**
AB titter	Numerical	69.25	–	–
AB titter binary	Numerical (0 = AB_titter<=10, 1 = AB_titter>10)	0.38	–	–
ACE/ARB	Categorical (yes, no)	0.25	–	–
Age	Numerical	56.87	–	–
Albumin	Numerical	2.59	4.4	4.4
ALP	Numerical	215.63	80	80
ALT	Numerical	162.82	20	20
AST	Numerical	221.28	20.5	20.5
BMI	Numerical	31.81	–	–
BNP	Numerical	805.52	100	100
CKD	Categorical	0.25	–	–
COPD	Categorical (yes, no)	0.12	–	–
COVID-19 infection	Categorical (yes, no)	0.50	–	–
CRP	Numerical	111.85	10	10
D-dimer	Numerical	4.20	0.5	0.5
DM	Categorical (yes, no)	0.38	–	–
ECMO	Categorical (yes, no)	0.25	–	–
Ferritin	Numerical	1017.39	180	159
Fibrinogen	Numerical	575.93	300	300
FIO2	Numerical	59.83	21	21
HbA1c	Numerical	6.93	5.7	5.7
HTN	Categorical (yes, no)	0.80	–	–
LDH	Numerical	542.74	219	219
LVEDD	Numerical	5.06	4.55	4.55
LVEF	Numerical	45.67	60	60
Mechanical ventilation	Categorical (yes, no)	0.33	–	–
O2Sat	Numerical	93.94	97.5	97.5
PCO2	Numerical	50.37	40	40
PEEP	Numerical	9.87	6.5	6.5
Platelets	Numerical	209.03	300	300
PO2	Numerical	93.94	87.5	87.5
RV dysfunction	Categorical (yes, no)	0.49	0	0
Sex	Categorical (M = male, F = female)	0.60	–	–
Survival outcome	Categorical (S = survived, D = deceased)	0.70	–	–
Troponin I	Numerical	1.36	0.02	0.02

#### 3.2.1 Prediction of patient’s survival

For predicting the patient’s survival, all parameters except mechanical ventilation, ECMO, and PEEP were used to train the ML model. The rationale for this approach was to find parameters that would allow prediction for survival even before the patient might clinically demonstrate the need for mechanical ventilation, which would also enable better planning in the hospital setting. After applying the feature selection procedure, the best models *SURiEx11* and *SUAiEx10* were created using reference and average imputation of missing data, respectively. These models utilized nine common parameters: Ferritin, PCO2, AB titer, Platelets, Albumin, AB titer binary, O2Sat, COVID-19 infection, and LDH. *SURiEx11* also included CRP and FIO2, while *SUAiEx10* included ACE/ARB.

Additionally, we searched for the model using just a few features with good prediction efficacy. We generated models *SURiEx3* and *SUAiEx2*, for reference and average imputation, respectively, which used just three parameters: Ferritin, CRP, and PCO2; or two parameters: Ferritin and PCO2, respectively.

The models’ prediction efficacy assessed using 5-fold cross-validations with 50 iterations of random splits is provided in Table 4. For most of the four models, AUC is above 0.87, ACC is greater than 0.80, and *F* measures are >0.85.

**Table 4:** Efficacy of models for predicting patient survival.

	**SURiEx11**	**SUAiEx10**	**SURiEx3**	**SUAiEx2**
AUC	0.8861	0.8839	0.8463	0.8737
AUCPR	0.9534	0.9537	0.8912	0.9374
ACC	0.8017	0.8083	0.8190	0.8050
*F* measure	0.8535	0.8637	0.8720	0.8584
Precision	0.8831	0.8622	0.8650	0.8431
Specificity	0.7444	0.6722	0.6767	0.6178
MCC	0.5505	0.5421	0.5644	0.5085
Sensitivity	0.8262	0.8667	0.8800	0.8752

The variable importances of selected features for models *SURiEx11*, *SUAiEx10*, *SURiEx3*, and *SUAiEx2* are presented in Supplementary Tables S3–S6.

#### 3.2.2 Prediction of whether a patient will require mechanical ventilation

For the generation of ML models for the prediction of whether a patient will require mechanical ventilation, we used three approaches, depending on the set of features used for training the ML models:

1. Extended set of features, which contains all parameters excluding survival outcome, ECMO, and PEEP. Survival outcome as the final event should not be used as a prediction feature, while mechanical ventilation is often used in combination with ECMO to manage the respiratory aspects of a condition (Combes et al., 2018). Additionally, PEEP is directly related to patients on mechanical ventilation (Carpio and Mora, 2024). After applying the feature selection procedure, we obtained the two best models in terms of prediction efficacy, depending on the imputation values of missing data, namely *MVRiEx9* and *MVAiEx7*, for reference values imputation and average values imputation, respectively. Both models used seven following parameters: PCO2, CRP, Platelets, Albumin, ALP, Fibrinogen, and AB titer, while model *MVRiEx9* used two more: FIO2 and Ferritin.
2. Medium set of features, which contains all parameters excluding survival outcome, ECMO, PEEP, PCO2, PO2, O2Sat, and FIO2. The parameters related to oxidation were excluded here due to their biased measurements, i.e., the most missing values were for the patients not on mechanical ventilation. The best models, *MVRiMed7* and *MVAiMed10*, for reference and average imputation, respectively, used the following seven parameters: CRP, Albumin, Fibrinogen, Platelets, ALP, AB titer, and AB titer binary, while *MVAiMed10* model used three more: COVID-19 infection, ACE/ARB, and D dimer.
3. A limited set of features, which contains all parameters excluding survival outcome, ECMO, PEEP, PCO2, PO2, O2Sat, FIO2, and CRP, since CRP was mostly measured only for patients on mechanical ventilation. The best models *MVRiLim7* and *MVAiLim9*, for reference and average imputation, respectively, used the following seven parameters: Albumin, Fibrinogen, Platelets, ALP, AB titer, D dimer, and AB titer binary, while *MVAiLim9* used two more: COVID-19 infection and ACE/ARB. The models’ prediction efficacy, evaluated using 5-fold cross-validations with 50 iterations of random splits, is given in Table 5. For all six models, AUC is above 0.95, ACC greater than 0.90, and *F* measure >0.85.

**Table 5:** Efficacy of models in predicting whether a patient will require mechanical ventilation.

	**MVRiEx9**	**MVAiEx7**	**MVRiMed7**	**MVAiMed10**	**MVRiLim7**	**MVAiLim9**
AUC	0.9713	0.9793	0.9559	0.9596	0.9669	0.9775
AUCPR	0.9575	0.9605	0.9369	0.9447	0.9494	0.9658
ACC	0.9280	0.9223	0.9117	0.9139	0.9080	0.9260
*F* measure	0.8870	0.8775	0.8563	0.8568	0.8606	0.8791
Precision	0.9263	0.9223	0.9333	0.9594	0.8696	0.9650
Specificity	0.9655	0.9645	0.9710	0.9826	0.9350	0.9845
MCC	0.8369	0.8235	0.8528	0.8067	0.7932	0.8340
Sensitivity	0.8530	0.8380	0.7930	0.7765	0.8540	0.8090

The variable importances of selected features for models *MVRiEx9*, *MVAiEx7*, *MVRiMed7*, *MVAiMed10*, *MVRiLim7*, and *MVAiLim9* are provided in Supplementary Tables S7–S12.

The comparison between different machine learning algorithms with the Ensemble model and the comparison between different selected numbers of features in the feature selection procedure is presented in the Supplementary Table S15 and Supplementary Figures S3, S4.

After combining various parameters to develop the most robust prediction model for our study’s outcomes, our findings demonstrated that a combination of parameters such as AB titer, use of ACE/ARB, Albumin, ALP, COVID-19 infection status, CRP, D-dimer, ECMO, Ferritin, Fibrinogen, FIO2, O2Sat, PCO2, and Platelets, proves to be the most powerful in evaluating both survival and the likelihood of requiring mechanical ventilation. More specifically, we found that the most effective set of parameters for predicting survival in our study group included Ferritin, PCO2, and CRP. When it came to predicting the probability of needing mechanical ventilation, a combination of Albumin, Fibrinogen, Platelets, ALP, AB titer, and D-dimer proved to be the most powerful set of parameters. Table 5 shows the high efficacy of predicting a patient’s likelihood of requiring mechanical ventilation.

The comparison of results between the statistical analyses, including ANOVA and t-test, and the findings from machine learning-based identification of important parameters showed very similar results (Supplementary Tables S13, S14). However, when examining the significant parameters for patient survival, the significant difference in Fibrinogen and Sex was identified by statistical analyses, while O2Sat, PCO2, CRP, and LDH were identified as important factors by machine learning. In the case of mechanical ventilation, statistical analyses identified Age, Ferritin, LVEDD, and LVEF as differentiating factors.

## 4 Discussion

In our study, we seek to discover potential markers of the severity of COVID-19 infection and factors that could improve survival in cardiovascular, specifically HF patients. Our goal is to carefully identify these factors and enable healthcare providers to implement a warning system in daily practice and identify patients likely to develop complications and need for mechanical ventilation. We believe that proper triaging and management of these patients can contribute to a better quality of care and more efficient utilization of ventilators, ICU beds, and general hospital capacity. Knowing that many medical facilities have limited capacities with staff, ventilators, ICU beds, or even a lack of ventilators, we believe models are needed that could better predict which patients are at higher risk for needing a higher level of care and which patients could be medically prioritized or transferred to another facility to match their medical needs better.

Our data confirmed that COVID-19 is associated with severe complications and a higher risk of needing mechanical ventilation, as well as with increased mortality risk among cardiovascular patients. Various patients’ characteristics, laboratory, and echocardiographic findings are shown to be associated with poor outcomes. Careful examination and evaluation of these parameters by healthcare providers could play a key role in the proper management of patients.

Since ferritin, fibrinogen, and platelets are coagulation markers, we hypothesize that these parameters played an important role in negatively affecting the survival of our study population by increasing the risk of a hypercoagulation state and thrombosis. Our study showed that decreased albumin levels play an important role in the assessment of the severity of COVID-19 and survival in cardiovascular patients, which correlates with findings from a previous study (Turcato et al., 2022).

There is a lot of controversial evidence about the use of ACE/ARBs in COVID-19 patients and their impact on clinical outcomes. A substantial cohort study conducted in England reported that the use of ACE/ARBs is associated with a decreased risk of COVID-19 disease and does not alter the risk of requiring intensive care unit (ICU) care (Hippisley-Cox et al., 2020). Nevertheless, a randomized clinical trial revealed that in severely ill patients hospitalized for COVID-19, the initiation of ACEI/ARBs did not lead to improvement; instead, it worsened clinical outcomes (Lawler et al., 2023). Our study data showed that our cardiovascular patients who were on ACE/ARB had a less likely chance of dying or having major complications that would require mechanical ventilation. Several previously proposed hypotheses hinge on the interaction between the viral spike protein and the ACE2 receptor, involving potential competition between the virus’s spike protein and drugs binding to ACE2 (Kumar et al., 2022). Furthermore, following the binding and potential activation, SARS-CoV-2 can induce downregulation of ACE2, leading to elevated concentrations of angiotensin II, which in turn can contribute to severe lung injury (Yehualashet and Belachew, 2020).

Various studies present conflicting perspectives on the role of ACE-2 in COVID-19. Some suggest that the availability of ACE-2 is directly correlated with the severe inflammatory response in COVID-19, while others propose that the free form of ACE2 may deactivate SARS-CoV-2 and prevent the virus from entering the lungs (Yehualashet and Belachew, 2020).

Another explanation of the survival benefit of ACE/ARBs could lie in better blood pressure control, knowing that hypertension is one of the factors that influence the severity of COVID-19. Our findings align with the clinical guidelines of the International Society of Hypertension, which state that there is no clear indication to discontinue the use of ACEI/ARBs in COVID-19 patients (European Society of Cardiology, 2020).

We identified that the most efficient combination of parameters for predicting survival within our study group consisted of Ferritin, PCO2, and CRP. When it came to predicting the likelihood of requiring mechanical ventilation, the combination of Albumin, Fibrinogen, Platelets, ALP, AB titer, and D-dimer emerged as the most potent set of parameters.

We believe that the incorporation of all these parameters in outcome risk calculations would significantly enhance predictive accuracy and effectiveness in assessing not only survival rates but also the probability of necessitating mechanical ventilation in our study group. In practical terms, this implies that healthcare professionals and researchers can rely on this predictive model to make more informed decisions regarding patient care, allowing for timely interventions, tailored treatment strategies, and improved patient outcomes.

We believe that the use of the combination of these parameters could represent a significant step forward, demonstrating the potential to revolutionize the way we approach risk assessment and treatment planning among a general cardiovascular patient population.

### 4.1 Limitations and future work

One of the limitations of our study was missing data for some patients, which we tried to minimize by excluding missing data, as well as replacing the missing data with normal and average values. Replacing the missing data with reference values could potentially lead to bias, but we justified that by the need to include some values when running the machine learning program and learning.

Another limitation is our small sample size of 60 cardiovascular patients. Thus, further studies that would include a larger sample size are encouraged. Moreover, it’s important to note that our study focused on a specific group of severely ill, hospitalized patients, and findings may vary from those in the broader cardiovascular patient population.

There are many justifications for conducting studies with small sample sizes in medical research (Leon et al., 2011; Indrayan and Mishra, 2021). Sample size may not be the main issue, but the real goal is to design and conduct a high-quality study, and analyze and interpret the results (Lenth, 2001; Bacchetti, 2013), as Matthews argued that the excessive emphasis on trial size can be counterproductive (Matthews, 1995). Studies with a small number of subjects can be quick to conduct, obtaining ethical and institutional approval is easier, it is often better to test a new research hypothesis, avoiding too many resources, it can be carried out in one center. However, the results need to be interpreted carefully and should be used to design larger confirmatory studies (Hackshaw, 2008; Indrayan and Mishra, 2021). There is the existence of many useful studies on small samples (Hansen and Fulton, 2000; Hatchell et al., 2021; Machado et al., 2021); some big discoveries started with case series (Gottlieb et al., 1981; CDC-Centers for Disease Control and Prevention, 1996). The small samples may be enough to show the existence of an effect but not for quantifying the effect (Anderson and Vingrys, 2001), while no single study based on a small or a large sample can be considered conclusive (Indrayan and Mishra, 2021).

In machine learning, the small train sample sizes pose significant challenges, including the risk of overfitting and reduced statistical power. To address these issues, we used an Ensemble model that combines multiple submodels to produce a single predictive outcome. We employed bagging, boosting, stacking, and regularization techniques to mitigate overfitting and improve generalizability (Dietterich, 2000; Friedman et al., 2010). Additionally, as a robust framework for the evaluation of ML predictors, we applied 5-fold stratified cross-validation (with 50 iterations of random splits) to reduce the variance of the performance estimates. This approach offers a more accurate assessment of model capabilities (Berrar, 2019). As one of the algorithms included in our Ensemble model, Deep Learning has been shown to be an effective algorithm in analyzing biomedical and health data (Miotto et al., 2018; Nasiri and Alavi, 2022). Incorporating ANOVA and t-tests statistical analyses with the ML feature selection process has been shown to be effective in identifying significant features (Zhou and Wang, 2007; Ding et al., 2014; Elssied et al., 2014; Abdulmohsin et al., 2021; Nasiri and Alavi, 2022). This enhancement improves the predictive power of ML models. By focusing on statistically significant features, the models are less prone to overfitting, more interpretable, and robust (Li et al., 2017).

For future work, we propose to evaluate markers for the severity of COVID-19 and for the prediction of survival, as well as the likelihood of needing mechanical ventilation in a population with preexisting CVD using AI models. This evaluation will enable us to create an AI-based model that could be used to better evaluate patients with preexisting CVD, assess their risk of mechanical ventilation, and manage their condition according to the associated risk. Given the limited number of hospital beds and mechanical ventilators, the AI model should accurately predict which patients have a higher risk for complications requiring more resources from medical facilities, such as ventilators and ICU beds. The ultimate goal is to enable more efficient and rapid medical decisions to improve the future management of Coronavirus in cardiovascular patients, which would hopefully also improve survival.

We also propose to expand our research goal and focus on cardiovascular patients in general, regardless of their COVID-19 status. Creating an app that incorporates individual patient parameters would allow for the calculation of a cardiovascular patient’s risk of mortality, as well as the risk of severe complications with a higher likelihood of needing mechanical ventilation. This would be an important tool for healthcare providers to assess patients’ general health status, determine risks of developing outcomes, and provide appropriate management and treatment.

## 5 Conclusion

The results of our study confirmed that COVID-19 is associated with a severe outcome. We identified that increased Ferritin, Fibrinogen, Platelets, and decreased Albumin have a negative impact on patients’ survival, while patients on ACE/ARB had a lower chance of dying or needing mechanical ventilation. The AI-based prediction models revealed that Ferritin, PCO2, and CRP were the most efficient combination of parameters for predicting patients’ survival, while the parameters Albumin, Fibrinogen, Platelets, ALP, AB titer, and D-dimer were the most efficient combination for predicting patients’ likelihood of requiring mechanical ventilation.

We believe our study findings would be useful for risk stratification and to guide clinical decisions in cardiovascular patients with COVID-19. A thorough assessment of the patient’s demographic data, medical history, as well as laboratory and echocardiographic findings is essential. Additionally, the AI-based prediction model could more accurately and rapidly identify patients at the highest risk of complications in general, facilitating the triage and redistribution of medical services to those in the greatest need. Our long-term goal is to improve the overall survival of cardiovascular patients by predicting their likelihood of severe complications or death and managing these patients accordingly. Furthermore, we believe that the use of the most potent combination of parameters in our prediction model could significantly advance our approach to risk assessment and treatment planning not only in our study group but also among the general population of cardiovascular patients.

## Data availability statement

The original contributions presented in the study are included in the article/Supplementary material, further inquiries can be directed to the corresponding authors.

## Ethics statement

The studies involving humans were approved by Committee for the Protection of Human Subjects University of Texas Health Science in Houston (UTHealth Houston) 7000 Fannin St, Suite 1840 Houston, Texas 77,030. The studies were conducted in accordance with the local legislation and institutional requirements. The ethics committee/institutional review board waived the requirement of written informed consent for participation from the participants or the participants’ legal guardians/next of kin because Minimal risk during the research that was not greater than those ordinarily encountered in during the required of routine physical or medical examinations or tests. All tests performed were part of the standard care and only de-identified samples were used.

## Author contributions

SM: Writing – original draft, Writing – review & editing, Data curation, Investigation, Resources, Validation. IG: Investigation, Writing – review & editing. RR: Writing – review & editing, Validation. SP: Writing – review & editing, Conceptualization, Methodology, Project administration, Writing – original draft. VP: Methodology, Writing – original draft, Writing – review & editing, Formal analysis, Software, Visualization.
