# Supplementary material for: Risk stratification and prediction of severity of COVID-19 infection in patients with preexisting cardiovascular disease
Source: Front Microbiol. 2024 Jul 25;15:1422393. doi: 10.3389/fmicb.2024.1422393 (PMC11306936; doi:10.3389/fmicb.2024.1422393)
Supplement: Supplementary file 2 [file Presentation_1.PDF]

Supplementary Material

CONTENT

1 ANOVA and t-test analyses………………………………………………………….. 2

1.1 ANOVA and t-test analyses between patient’s survival and each parameter……..…………………………………… 2

1.2 ANOVA and t-test analyses between patient’s mechanical ventilation and each parameter…………………………..... 6

2 Machine Learning in identification of important parameters……………………..……………………………….…… 10

2.1 Performance metrics………………………………………….…………………………………………..…... 10

2.2 Prediction of patient’s survival……………………………….……………….………………………..……. 11

2.3 Prediction if patient will end up on mechanical ventilation……………….…..…………………………………….…… 13

3 Tables of parameters and their importances……………………………………………………………….…… 17

4 Comparison of machine learning algorithms with the ensemble model……….……………………………………. 21

5 Feature selection procedure and comparison between different selected numbers of features……………………………. 22

6 REFERENCES……………………………………………………………………………………………..… 23

1 ANOVA and t-test analyses

1.1 ANOVA and t-test analyses between patient’s survival and each parameter

We performed the ANOVA and two-tailed t-test analyses of patients’ survival outcomes and each of the parameters by:

1) Excluding pairs with missing data;
2) Replacing missing data with average values; and
3) Replacing missing data with reference values.

The results are presented in a tabular format in **Supplementary Table 1** and also as a graphical representation in **Supplementary Figure 1** using P-values. The statistically significant parameters with P-values < 0.05 are marked in orange.

Supplementary Table 1. The ANOVA and t-test analyses between patients’ survival and each parameter

The column names are:
AN_F_ep – ANOVA F-Value excluding missing pairs;
AN_P_ep – ANOVA P-value excluding missing pairs;
TT_t_ep – t-test t-stat excluding missing pairs;
TT_p_ep – t-test p-value two-tail excluding missing pairs;
AN_F_ep – ANOVA F-Value replace missing data with averages;
AN_P_ep – ANOVA P-value replace missing data with averages;
TT_t_ep – t-test t-stat replace missing data with averages;
TT_p_ep – t-test p-value two-tail replace missing data with averages;
AN_F_ep – ANOVA F-Value replace missing data with reference values;
AN_P_ep – ANOVA P-value replace missing data with reference values;
TT_t_ep – t-test t-stat replace missing data with reference values;
TT_p_ep – t-test p-value two-tail replace missing data with reference values.

The ANOVA and t-test P values less than 0.05 are colored in orange. The statistically significant parameters, with all their P values < 0.05, are also colored in orange.

Parameter	AN_F_ep	AN_P_ep	TT_t_ep	TT_p_ep	AN_F_ia	AN_P_ia	TT_t_ia	TT_p_ia	AN_F_ir	AN_P_ir	TT_t_ir	TT_p_ir
AB titter	9.462	3.20E-03	2.263	0.0354	9.462	3.20E-03	2.263	0.0354	9.462	3.20E-03	2.263	0.0354
AB titter binary	22.79	1.27E-05	4.717	4.71E-05	22.79	1.27E-05	4.717	4.71E-05	22.79	1.27E-05	4.717	4.71E-05
ACE/ARB	9.667	2.91E-03	-4.773	2.32E-05	9.667	2.91E-03	-4.773	2.32E-05	9.667	2.91E-03	-4.773	2.32E-05
Age	0.0642	0.8008	0.2429	0.8097	0.0642	0.8008	0.2429	0.8097	0.0642	0.8008	0.2429	0.8097
Albumin	23.64	9.23E-06	-4.949	2.06E-05	23.64	9.23E-06	-4.949	2.06E-05	23.64	9.23E-06	-4.949	2.06E-05
ALP	1.929	0.1703	1.35	0.1869	1.948	0.1681	1.349	0.1874	2.045	0.1581	1.383	0.177
ALT	1.399	0.2417	0.8461	0.4082	1.399	0.2417	0.8461	0.4082	1.399	0.2417	0.8461	0.4082
AST	1.534	0.2205	0.8718	0.3945	1.534	0.2205	0.8718	0.3945	1.534	0.2205	0.8718	0.3945
BMI	0.7281	0.397	-0.9554	0.3448	0.7281	0.397	-0.9554	0.3448	0.7281	0.397	-0.9554	0.3448
BNP	0.5183	0.4757	-1.148	0.2582	0.4821	0.4903	-1.019	0.3133	0.7144	0.4015	-1.248	0.2182
CKD	0.9355	0.3375	0.9083	0.3714	0.9355	0.3375	0.9083	0.3714	0.9355	0.3375	0.9083	0.3714
COPD	7.45E-03	0.9315	-0.0868	0.9314	7.45E-03	0.9315	-0.0868	0.9314	7.45E-03	0.9315	-0.0868	0.9314
COVID-19 infection	20.3	3.26E-05	5.243	3.75E-06	20.3	3.26E-05	5.243	3.75E-06	20.3	3.26E-05	5.243	3.75E-06
CRP	0.2664	0.61	0.5184	0.6085	0.3243	0.5712	0.4572	0.6521	11.6	1.20E-03	2.966	6.64E-03
D-dimer	1.424	0.2389	1.187	0.2432	1.597	0.2113	1.17	0.252	4.457	0.0391	2.005	0.0544
DM	0.3954	0.532	0.6154	0.5428	0.3954	0.532	0.6154	0.5428	0.3954	0.532	0.6154	0.5428
ECMO	15.74	2.03E-04	3.34	2.82E-03	15.74	2.03E-04	3.34	2.82E-03	15.74	2.03E-04	3.34	2.82E-03
Ferritin	16.26	2.28E-04	3.281	4.30E-03	17.43	1.01E-04	2.879	9.97E-03	25.49	4.70E-06	3.42	3.11E-03
Fibrinogen	5.597	0.0225	2.189	0.0376	6.158	0.016	2.086	0.0482	11.49	1.26E-03	2.959	6.73E-03
FIO2	0.7206	0.4034	0.8523	0.4018	0.8711	0.3545	0.7518	0.4602	15.93	1.87E-04	3.507	1.74E-03
HbA1c	0.0361	0.8505	-0.2162	0.8305	0.0387	0.8448	-0.2232	0.8244	3.40E-04	0.9854	-0.0207	0.9836
HTN	0.9553	0.3324	-0.9016	0.3752	0.9553	0.3324	-0.9016	0.3752	0.9553	0.3324	-0.9016	0.3752
LDH	0.3829	0.5402	0.5777	0.5696	0.4592	0.5007	0.4902	0.6296	2.203	0.1431	1.086	0.291

LVEDD	2.106	0.1526	-1.462	0.1527	2.211	0.1424	-1.462	0.1539	1.627	0.2072	-1.263	0.2157
LVEF	0.499	0.4829	0.7197	0.4766	0.5143	0.4762	0.7205	0.4763	0.2796	0.599	0.5356	0.5958
Mechanical Ventilation	54	7.61E-10	6.896	1.65E-07	54	7.61E-10	6.896	1.65E-07	54	7.61E-10	6.896	1.65E-07
O2Sat	2.995	0.0916	-1.621	0.1184	3.532	0.0652	-1.351	0.1927	9.648	2.93E-03	-2.249	0.0366
PCO2	1.184	0.2845	1.09	0.2838	1.428	0.237	0.9844	0.3354	7.525	8.08E-03	2.286	0.0318
PEEP	0.0146	0.905	-0.1274	0.9006	0.0156	0.9011	-0.0899	0.9293	12.07	9.74E-04	2.718	0.0129
Platelets	8.22	5.80E-03	-2.986	5.07E-03	8.294	5.56E-03	-2.988	5.09E-03	8.696	4.59E-03	-3.066	4.15E-03
PO2	1.57E-03	0.9687	0.0396	0.9686	1.91E-03	0.9653	0.0362	0.9715	0.0939	0.7604	0.2538	0.802
RV dysfunction	7.82E-03	0.9299	0.0879	0.9305	8.06E-03	0.9288	0.0878	0.9306	0.1112	0.74	0.3306	0.7431
Sex	6.246	0.0153	2.791	7.87E-03	6.246	0.0153	2.791	7.87E-03	6.246	0.0153	2.791	7.87E-03
Troponin I	0.0875	0.7689	-0.3412	0.7347	0.0971	0.7565	-0.3638	0.7176	8.63E-03	0.9263	-0.1086	0.9139

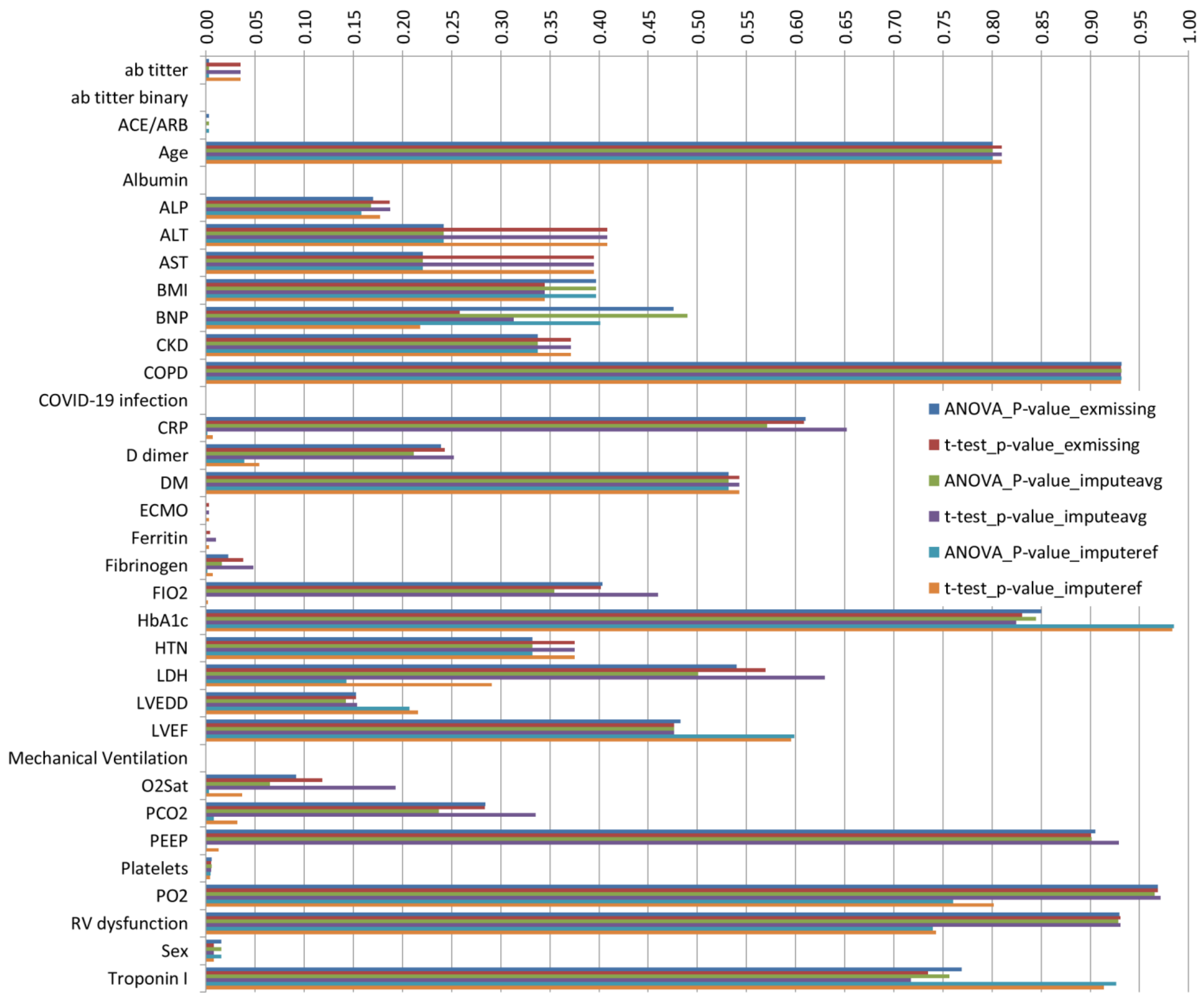

Supplementary Figure 1. P values of each parameter were compared with patient's survival using the ANOVA and t-test analyses

1.2 ANOVA and t-test analyses between patient's mechanical ventilation and each parameter

We completed the ANOVA and two-tailed t-test analyses for each parameter to determine if the patient will require mechanical ventilation. The following steps were taken:

1) Excluding pairs with missing data;
2) Replacing missing data with average values; and
3) Replacing missing data with reference values.

The results are presented in a tabular format in **Supplementary Table 2**. The parameters that showed statistical significance, with P-values < 0.05, are marked in orange. Additionally, the significance of the parameters is visually represented in **Supplementary Figure 2** using P-values.

Supplementary Table 2. ANOVA and t-test analyses between patient's mechanical ventilation and each parameter

The column names are:
AN_F_ep – ANOVA F-Value excluding missing pairs;
AN_P_ep – ANOVA P-value excluding missing pairs;
TT_t_ep – t-test t-stat excluding missing pairs;
TT_p_ep – t-test p-value two-tail excluding missing pairs;
AN_F_ep – ANOVA F-Value replace missing data with averages;
AN_P_ep – ANOVA P-value replace missing data with averages;
TT_t_ep – t-test t-stat replace missing data with averages;
TT_p_ep – t-test p-value two-tail replace missing data with averages;
AN_F_ep – ANOVA F-Value replace missing data with reference values;
AN_P_ep – ANOVA P-value replace missing data with reference values;
TT_t_ep – t-test t-stat replace missing data with reference values;
TT_p_ep – t-test p-value two-tail replace missing data with reference values.

The ANOVA and t-test P-values less than 0.05 are colored in orange. The statistically significant parameters with the majority of their P-values < 0.05 are also colored in orange.

Parameter	AN_F_ep	AN_P_ep	TT_t_ep	TT_p_ep	AN_F_ia	AN_P_ia	TT_t_ia	TT_p_ia	AN_F_ir	AN_P_ir	TT_t_ir	TT_p_ir
AB titter	20.90	2.58E-05	-3.391	2.82E-03	20.90	2.58E-05	-3.391	2.82E-03	20.90	2.58E-05	-3.391	2.82E-03
AB titter binary	33.66	2.89E-07	-5.676	1.88E-06	33.66	2.89E-07	-5.676	1.88E-06	33.66	2.89E-07	-5.676	1.88E-06
ACE/ARB	11.60	1.20E-03	4.837	2.09E-05	11.60	1.20E-03	4.837	2.09E-05	11.60	1.20E-03	4.837	2.09E-05
Age	5.339	0.0244	2.314	0.0261	5.339	0.0244	2.314	0.0261	5.339	0.0244	2.314	0.0261
Albumin	43.59	1.36E-08	6.793	3.18E-08	43.59	1.36E-08	6.793	3.18E-08	43.59	1.36E-08	6.793	3.18E-08
ALP	16.86	1.30E-04	-2.932	8.52E-03	16.97	1.22E-04	-2.906	9.00E-03	17.36	1.04E-04	-2.937	8.42E-03
ALT	4.587	0.0364	-1.551	0.1366	4.587	0.0364	-1.551	0.1366	4.587	0.0364	-1.551	0.1366
AST	3.854	0.0544	-1.399	0.1777	3.854	0.0544	-1.399	0.1777	3.854	0.0544	-1.399	0.1777
BMI	0.1112	0.7400	-0.3587	0.7214	0.1112	0.7400	-0.3587	0.7214	0.1112	0.7400	-0.3587	0.7214
BNP	0.6072	0.4404	1.241	0.2222	0.5330	0.4683	1.003	0.3211	1.027	0.3150	1.401	0.1681
CKD	1.589	0.2125	1.364	0.1791	1.589	0.2125	1.364	0.1791	1.589	0.2125	1.364	0.1791
COPD	1.278	0.2629	1.317	0.1934	1.278	0.2629	1.317	0.1934	1.278	0.2629	1.317	0.1934
COVID-19 infection	27.29	2.48E-06	-5.964	2.04E-07	27.29	2.48E-06	-5.964	2.04E-07	27.29	2.48E-06	-5.964	2.04E-07
CRP	10.67	2.96E-03	-3.454	1.99E-03	9.838	2.68E-03	-2.601	0.0155	48.83	3.08E-09	-5.537	1.28E-05
D-dimer	12.36	1.00E-03	-3.083	5.32E-03	13.19	5.98E-04	-2.747	0.0120	22.23	1.56E-05	-3.578	1.75E-03
DM	1.719	0.1950	-1.277	0.2099	1.719	0.1950	-1.277	0.2099	1.719	0.1950	-1.277	0.2099
ECMO	116.0	1.85E-15	-7.550	3.92E-07	116.0	1.85E-15	-7.550	3.92E-07	116.0	1.85E-15	-7.550	3.92E-07
Ferritin	17.02	1.71E-04	-3.638	1.68E-03	17.66	9.22E-05	-3.098	5.59E-03	27.20	2.56E-06	-3.761	1.25E-03
Fibrinogen	23.22	1.75E-05	-4.452	1.26E-04	23.57	9.48E-06	-4.063	4.16E-04	35.98	1.37E-07	-5.042	3.19E-05
FIO2	5.366	0.0284	-2.353	0.0278	5.381	0.0239	-1.940	0.0637	37.13	9.54E-08	-5.135	2.47E-05
HbA1c	0.8912	0.3522	-0.8710	0.3943	0.9621	0.3307	-0.8496	0.4030	1.573	0.2148	-1.084	0.2880
HTN	4.387	0.0406	1.851	0.0747	4.387	0.0406	1.851	0.0747	4.387	0.0406	1.851	0.0747
LDH	3.574	0.0672	-1.891	0.0745	3.980	0.0508	-1.435	0.1671	8.782	4.41E-03	-2.113	0.0478

LVEDD	4.854	0.0320	2.306	0.0258	5.049	0.0285	2.324	0.0251	3.994	0.0504	2.083	0.0433
LVEF	4.732	0.0339	-2.338	0.0236	4.848	0.0317	-2.357	0.0227	3.839	0.0549	-2.116	0.0397
O2Sat	3.803	0.0586	1.950	0.0598	4.247	0.0438	1.652	0.1119	11.73	1.13E-03	2.746	0.0114
Survive outcome	54.00	7.61E-10	6.254	1.27E-06	54.00	7.61E-10	6.254	1.27E-06	54.00	7.61E-10	6.254	1.27E-06
PCO2	8.522	6.28E-03	-2.932	6.30E-03	8.816	4.34E-03	-2.577	0.0157	25.08	5.45E-06	-4.228	2.63E-04
PEEP	2.032	0.1688	-1.978	0.0878	1.296	0.2596	-0.8370	0.4124	38.78	5.74E-08	-4.495	2.28E-04
Platelets	10.02	2.49E-03	3.317	1.84E-03	10.09	2.39E-03	3.321	1.84E-03	10.59	1.90E-03	3.406	1.44E-03
PO2	0.1838	0.6710	0.4225	0.6758	0.2072	0.6507	0.3984	0.6934	0.0289	0.8656	0.1491	0.8826
RV dysfunction	0.4140	0.5226	-0.6414	0.5250	0.4250	0.5170	-0.6409	0.5256	0.8206	0.3688	-0.8999	0.3739
Sex	1.234	0.2712	-1.137	0.2621	1.234	0.2712	-1.137	0.2621	1.234	0.2712	-1.137	0.2621
Troponin I	0.1073	0.7449	0.3653	0.7169	0.1180	0.7324	0.3991	0.6914	6.11E-03	0.9380	0.0909	0.9279

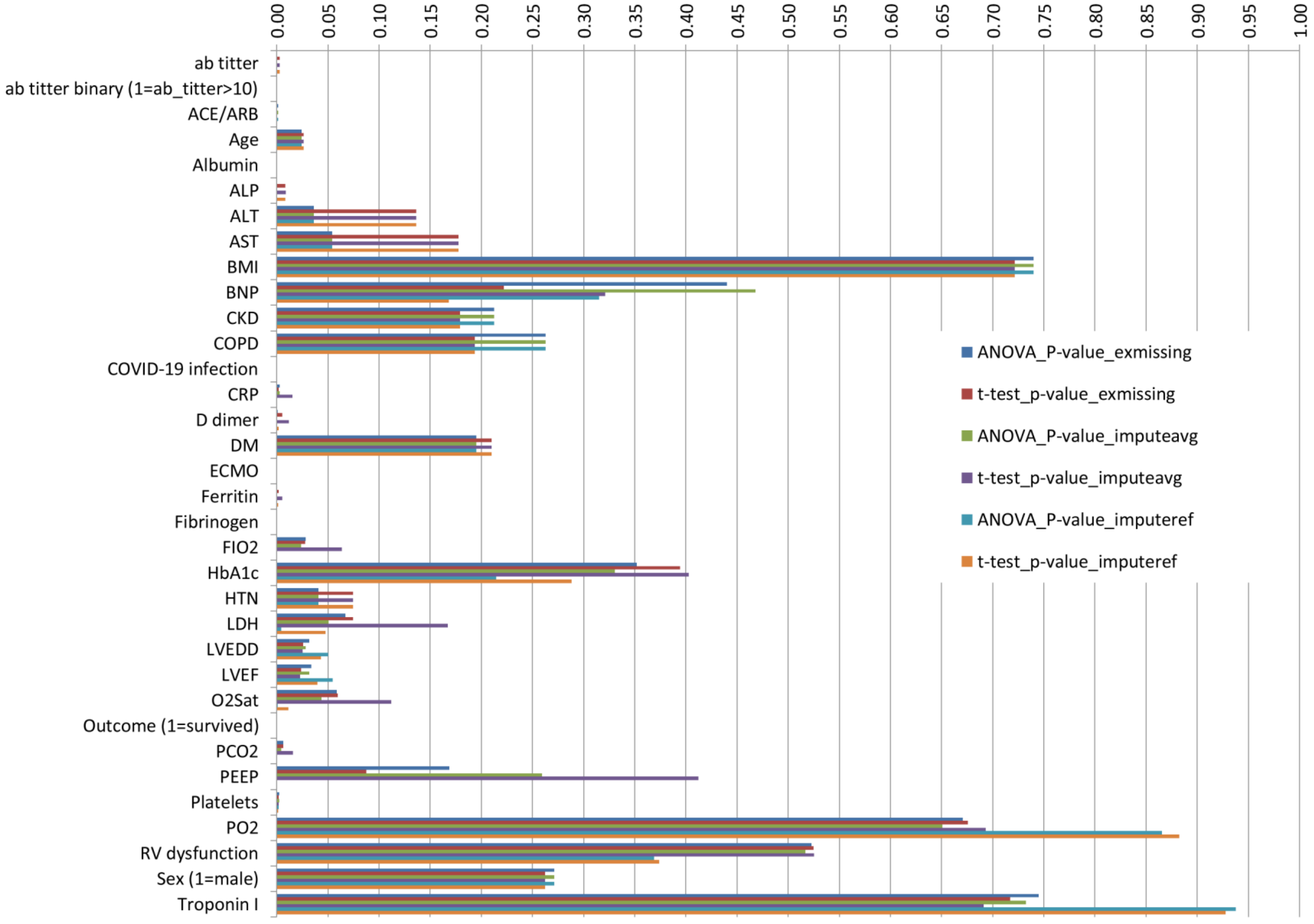

Supplementary Figure 2. P values of each parameter were compared with the patient's mechanical ventilation outcome using ANOVA and t-test analyses

2 Machine Learning in identification of important parameters

To establish the association (correlation) between individual parameters and predictive outcomes, we used machine learning techniques..

2.1 Performance metrics

For the evaluation of the generated machine learning predictors the following performance metrics were used: the area under the receiver operating characteristic curve (AUC), area under the precision-recall plots (AUCPR), accuracy (ACC), precision, sensitivity (or recall), specificity, F score, and the Matthews correlation coefficient (MCC).

Evaluation measures were calculated as:

$$ACC = \frac{TP + TN}{TP + TN + FP + FN}$$

$$precision = \frac{TP}{TP + FP}$$

$$recall = \frac{TP}{TP + FN}$$

$$specificity = \frac{TN}{TN + FP}$$

$$F\ score = \frac{2 * precision * recall}{precision + recall}$$

$$MCC = \frac{TP * TN - FP * FN}{\sqrt{(TP + FP)(TP + FN)(TN + FP)(TN + FN)}}$$

where TP represents True Positives; FP - False Positives; TN - True Negatives; FN - False Negatives.

The receiver operating characteristic (ROC) and Precision-Recall Curves were calculated using R package h2o.ai [LeDell et al. 2022].

2.2 Prediction of patient's survival

For prediction of patient's survival we generated two models *SURiEx11* and *SUAiEx10* using reference and average imputation of missing data respectively. Furthermore, using only a few features with good prediction efficacy, we created two models *SURiEx3* and *SUAiEx2* using reference and average imputation respectively.

The list of selected features with their variable importances used in models *SURiEx11*, *SUAiEx10*, *SURiEx3* and *SUAiEx2* is presented in **Supplementary Tables 3-6**.

Supplementary Table 3. Variable importances for model *SURiEx11*.

**Variable**	**Scaled importance**
CRP	1.000000
Ferritin	0.743001
PCO2	0.695227
Platelets	0.591489
FIO2	0.425836
Albumin	0.378530
AB titter	0.330774

AB titter binary	0.293748
LDH	0.158718
COVID-19 infection	0.098493
O2Sat	0.060002

Supplementary Table 4. Variable importances for model *SUAiEx10*.

**Variable**	**Scaled importance**
Ferritin	1.000000
PCO2	0.937500
Platelets	0.937500
Albumin	0.833333
O2Sat	0.770833
COVID-19 infection	0.687500
AB titter	0.666667
AB titter binary	0.666667
LDH	0.666667
ACE/ARB	0.645833

Supplementary Table 5. Variable importances for model *SURiEx3*.

**Variable**	**Scaled importance**
Ferritin	1.000000

PCO2	0.771027
CRP	0.716154

Supplementary Table 6. Variable importances for model *SUAiEx2*.

**Variable**	**Scaled importance**
Ferritin	1.000000
PCO2	0.937500

2.3 Prediction if patient will end up on mechanical ventilation

For the generation of ML models to predict if a patient will end up on mechanical ventilation, we created models using different sets of features for training ML models:

1) Using an extended set of features, we created the models *MVRiEx9* and *MVAiEx7* for reference and average value imputation respectively,
2) Using a medium set of features, we generated the models *MVRiMed7* and *MVAiMed10* for reference and average imputation respectively,
3) Using a limited set of features, we obtained the models *MVRiLim7* and *MVAiLim9* for reference and average imputation respectively.

The variable importances of the selected features for the models *MVRiEx9*, *MVAiEx7*, *MVRiMed7*, *MVAiMed10*, *MVRiLim7* and *MVAiLim9* are presented in **Supplementary Tables 7-12**.

Supplementary Table 7. Variable importances for model *MVRiEx9*.

**Feature**	**Scaled importance**
PCO2	1.000000
CRP	0.956522
Platelets	0.891304
FIO2	0.869565
Albumin	0.782609
ALP	0.782609
Fibrinogen	0.782609
Ferritin	0.673913
AB titter	0.608696

Supplementary Table 8. Variable importances for model *MVAiEx7*.

**Feature**	**Scaled importance**
PCO2	1.000000
Platelets	0.956522
Fibrinogen	0.847826
Albumin	0.826087
ALP	0.826087
CRP	0.804348
AB titter	0.695652

Supplementary Table 9. Variable importances for model *MVRiMed7*.

**Variable**	**Scaled importance**
CRP	1.000000
Albumin	0.605312
Fibrinogen	0.519678
Platelets	0.516169
AB titter	0.515768
AB titter binary	0.376883
ALP	0.361064

Supplementary Table 10. Variable importances for model *MVAiMed10*.

**Variable**	**Scaled importance**
Albumin	1.000000
Platelets	1.000000
Fibrinogen	0.973684
CRP	0.868421
AB titter	0.789474
ALP	0.789474
AB titter binary	0.736842
ACE/ARB	0.710526
COVID-19 infection	0.710526
D dimer	0.631579

Supplementary Table 11. Variable importances for model *MVRiLim7*.

Variable	Scaled importance
Albumin	1.000000
Fibrinogen	0.972973
Platelets	0.891892
ALP	0.837838
AB titter	0.810811
D dimer	0.783784
AB titter binary	0.702703

Supplementary Table 12. Variable importances for model *MVAiLim9*.

Variable	Scaled importance
Albumin	1.000000
Fibrinogen	0.945946
Platelets	0.918919
ALP	0.837838
AB titter	0.810811
COVID-19 infection	0.756757
ACE/ARB	0.729730
AB titter binary	0.702703
D dimer	0.675676

3 Tables of parameters and their importances

The collected tables of all parameters, where important parameters are marked by an asterisk, are presented in **Supplementary Tables 13 and 14**. The importances obtained by each analysis are provided in a separate column. For ANOVA and two-tailed t-test analyses, excluding missing pairs, the statistically significant parameters are marked with an asterisk, while for each model generated by machine learning, the parameters included in the model are marked with an asterisk. The parameters which are excluded from the set in the training process, due to different approaches for the exclusion of features, as explained in sections 3.2.1 and 3.2.2 in the paper, are marked with an X. The important parameters related to the patient's survival outcome or whether the patient will require mechanical ventilation are provided in **Supplementary Table 13** and **Supplementary Table 14** respectively..

Supplementary Table 13. Statistically important variables related to the patient's survival outcome. Statistically important parameters or parameters selected as the final set of features in machine learning models are marked with an asterisk. The parameters excluded from the training set are marked with an X.

**Parameter**	**ANOVA_ep**	**t-test_ep**	**SURiEx11**	**SUAiEx10**	**SURiEx3**	**SUAiEx2**
AB titter	*	*	*	*		
AB titter binary	*	*	*	*		
ACE/ARB	*	*		*		
Age						
Albumin	*	*	*	*		
ALP						
ALT						
AST						
BMI						
BNP						
CKD						
COPD						
COVID-19 infection	*	*	*	*		

CRP			*		*	
D-dimer						
DM						
ECMO	*	*	x	x	x	x
Ferritin	*	*	*	*	*	*
Fibrinogen	*	*				
FIO2			*			
HbA1c						
HTN						
LDH			*	*		
LVEDD						
LVEF						
Mechanical Ventilation	*	*	x	x	x	x
O2Sat			*	*		
PCO2			*	*	*	*
PEEP			x	x	x	x
Platelets	*	*	*	*		
PO2						
RV dysfunction						
Sex	*	*				
Troponin I						

Supplementary Table 14. Statistically important variables related to the outcome of whether a patient will require mechanical ventilation. Statistically important parameters or parameters selected as the final set of features in machine learning models are marked with an asterisk. The parameters excluded from the training set are marked with an X.

**Parameter**	**ANOVA_ep**	**t-test_ep**	**MVRiEx9**	**MVAiEx7**	**MVRiMed7**	**MVAiMed10**	**MVRiLim7**	**MVAiLim9**
AB titter	*	*	*	*	*	*	*	*
AB titter binary	*	*			*	*	*	*
ACE/ARB	*	*				*		*
Age	*	*						
Albumin	*	*	*	*	*	*	*	*
ALP	*	*	*	*	*	*	*	*
ALT	*							
AST								
BMI								
BNP								
CKD								
COPD								
COVID-19 infection	*	*				*		*
CRP	*	*	*	*	*	*	x	x
D-dimer	*	*				*	*	*
DM								
ECMO	*	*	x	x	x	x	x	x
Ferritin	*	*	*					
Fibrinogen	*	*	*	*	*	*	*	*

FIO2	*	*	*		x	x	x	x
HbA1c								
HTN	*							
LDH								
LVEDD	*	*						
LVEF	*	*						
O2Sat					x	x	x	x
PCO2	*	*	*	*	x	x	x	x
PEEP			x	x	x	x	x	x
Platelets	*	*	*	*	*	*	*	*
PO2					x	x	x	x
RV dysfunction								
Sex								
Survival outcome	*	*	x	x	x	x	x	x
Troponin I								

4 Comparison of machine learning algorithms with the ensemble model

The comparison between different machine learning algorithms and the ensemble is presented using the example of model *MVRiLim7*. The performance statistics of each submodel, as well as the ensemble model, generated using 7 selected features, are presented in **Supplementary Figure 3**. The ensemble model outperforms other machine learning algorithms in terms of AUCPR, ACC, F-measure, MCC, and Sensitivity.

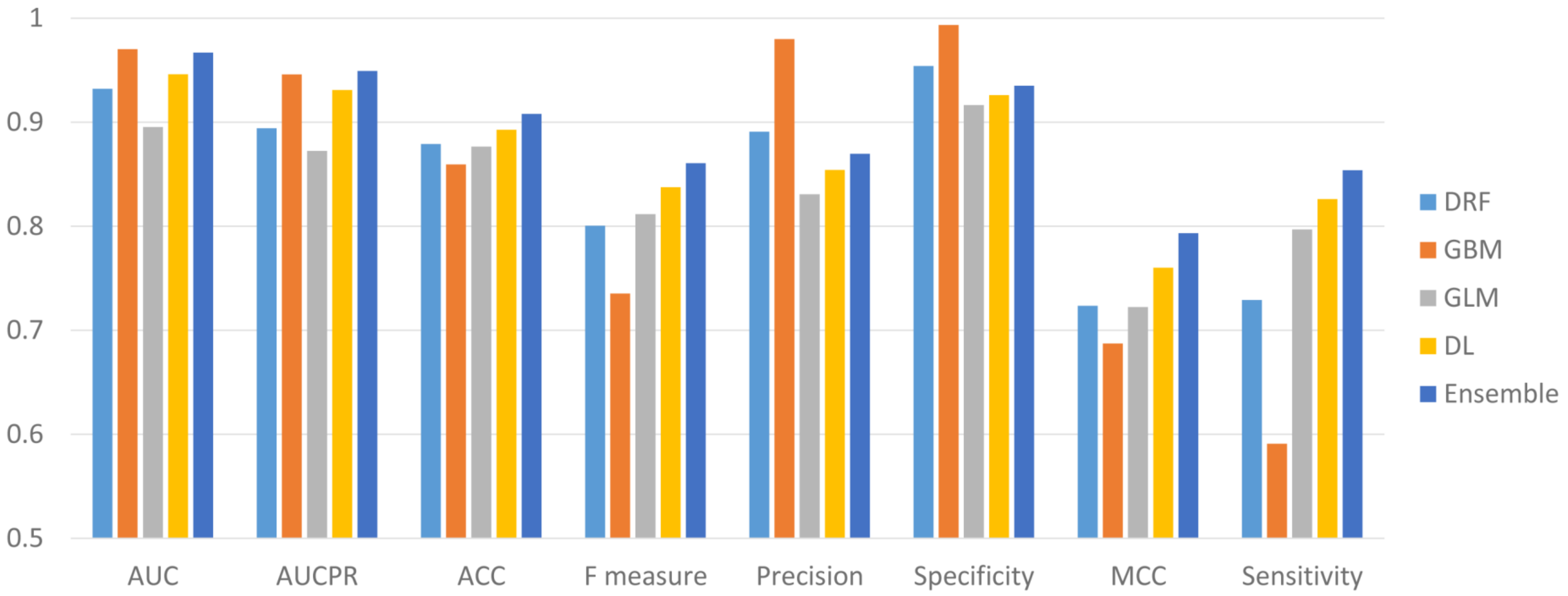

Supplementary Figure 3. Comparison between different ML algorithms and the ensemble of the *MVRiLim7* model.

5 Feature selection procedure and comparison between different selected numbers of features

The comparison between different numbers of selected features in the feature selection procedure is presented using the example of model *MVAiMed10*. The performance statistics of the Ensemble model for each number of selected features, ranging from 2 to 15, are presented in **Supplementary Figure 4**. The list of features used in the selection process is provided in **Supplementary Table 15**. The model with 10 selected features outperforms the others in terms of AUCPR, ACC, F measure, and MCC.

Supplementary Table 15. List of features used in the feature selection procedure, as presented in **Supplementary Figure 4**.

**Parameter**
Albumin
Platelets
Fibrinogen
CRP
AB titter
ALP
AB titter binary
ACE/ARB
COVID-19 infection
D dimer
Ferritin
LVEF
Age
RV dysfunction
CKD

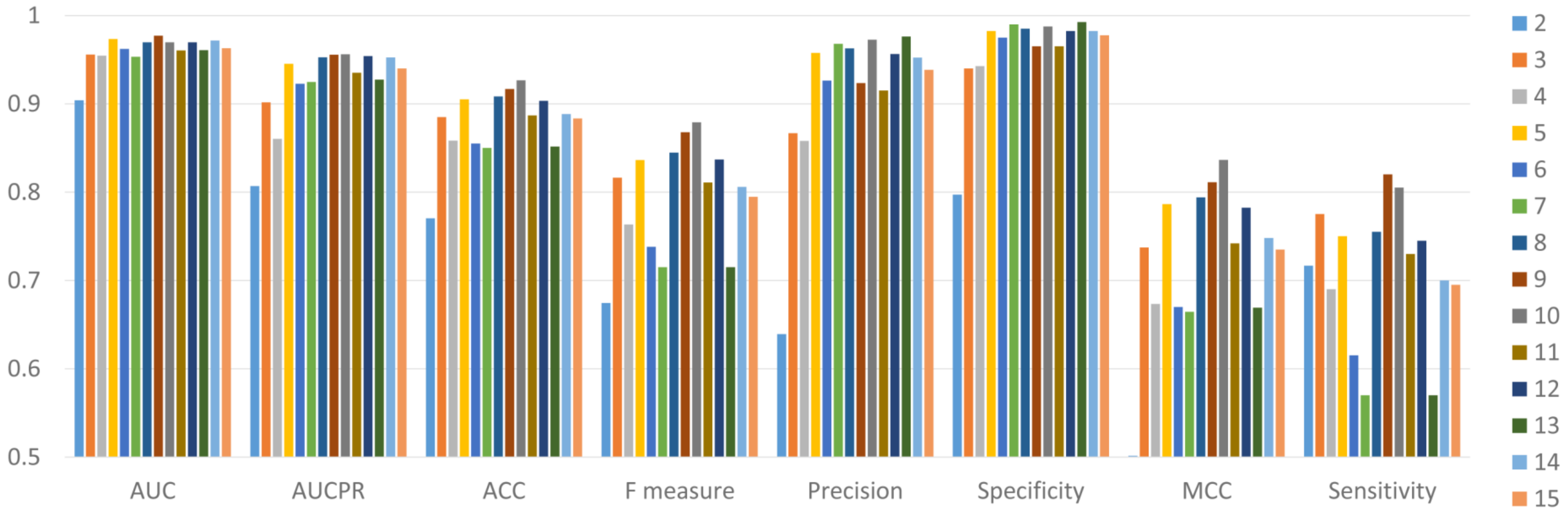

Supplementary Figure 4. Comparison between different numbers of features in the feature selection procedure for the generation of the *MVAiMed10* model.

REFERENCES

LeDell E, Gill N, Aiello S, Fu A, Candel A, Click C, Kraljevic T, Nykodym T, Aboyoun P, Kurka M, Malohlava M (2023). _h2o: R Interface for the 'H2O' Scalable Machine Learning Platform_. R package version 3.42.0.1, <https://github.com/h2oai/h2o-3>.
